# Multidomain Molecular Sensor Devices, Systems, and Algorithms for Improved Physiological Monitoring

**DOI:** 10.3390/mi16080900

**Published:** 2025-07-31

**Authors:** Lianna D. Soriano, Shao-Xiang Go, Lunna Li, Natasa Bajalovic, Desmond K. Loke

**Affiliations:** 1 College of Letters and Science, University of California, Berkeley, CA 94720, USA; 2 Department of Science, Mathematics and Technology, and The AI Mega Centre, Singapore University of Technology and Design, Singapore 487372, Singapore; 3 Thomas Young Centre and Department of Chemical Engineering, University College London, London WC1E 7JE, UK

**Keywords:** biomarkers, molecular sensors, machine learning, healthcare physiological monitoring, precision medicine

## Abstract

Molecular sensor systems, e.g., implantables and wearables, provide extensive health-related monitoring. Glucose sensor systems have historically prevailed in wearable bioanalysis applications due to their continuous and reliable glucose monitoring, a feat not yet accomplished for other biomarkers. However, the advancement of reagentless detection methodologies may facilitate the creation of molecular sensor systems for multiple analytes. Improving the sensitivity and selectivity of molecular sensor systems is also crucial for biomarker detection under intricate physiological circumstances. The term multidomain molecular sensor systems is utilized to refer, in general, to both biological and chemical sensor systems. This review examines methodologies for enhancing signal amplification, improving selectivity, and facilitating reagentless detection in multidomain molecular sensor devices. The review also analyzes the fundamental components of multidomain molecular sensor systems, including substrate materials, bodily fluids, power, and decision-making units. The review article further investigates how extensive data gathered from multidomain molecular sensor systems, in conjunction with current data processing algorithms, facilitate biomarker detection for precision medicine.

## 1. Introduction

**Figure 1:**
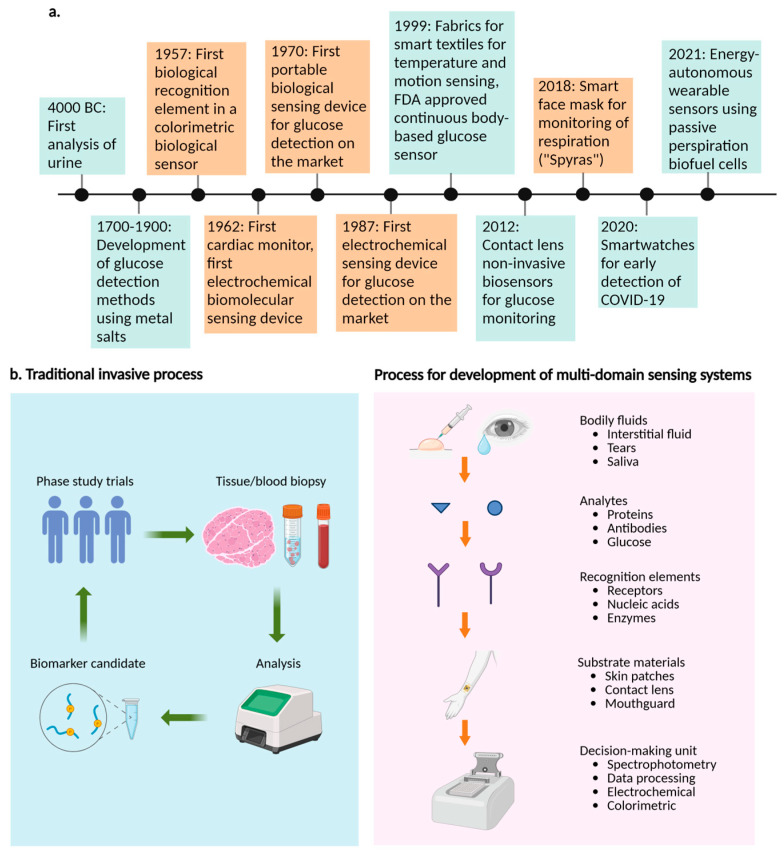
Chronological list of significant advancements in sensor systems’ development and biomarker identification facilitated by multidomain molecular sensor systems. (**a**) Advancements in molecular sensor systems for healthcare monitoring have been driven by data analysis, electronics, bioengineering, materials science, and telecommunication technologies [1,14,15,16,17,18,19,20,21,22,23]. The growing interest in healthcare physiological monitoring has led to cost reductions, enabling widespread adoption of these sensor systems globally. This has enabled continuous monitoring on a large scale. Additionally, advancements in fabrication techniques have allowed for increased complexity at smaller sizes, enabling molecular sensor systems to be integrated into personal technologies. (**b**) Precision medicine utilizes traditional blood or tissue biopsy techniques for biomarker detection, and the process for creating multidomain molecular sensor systems to detect biomarkers. Figure created with BioRender (BioRender.com).

The study of molecular sensor systems is an expanding interdisciplinary research area that integrates computational data analytics, medicine, physics, biology, chemistry, and engineering to monitor physiological conditions in real-time (Figure 1a) [1,2,3,4]. Despite advancements in portability, signal strength, and targeting specific analytes, there is still a gap between the number of traditional molecular sensor systems developed in laboratories and those successfully brought to the commercial market. Glucose sensor systems utilize specialized enzymes, viz., glucose dehydrogenase and glucose oxidase, to generate enhanced responses through high catalytic turnover [1,5]. However, other conventional types of nucleic acids, proteins, and small molecules struggle to achieve this continuous response. Body-based molecular sensor systems, e.g., implantables and wearables, require continuous-sensor mechanisms similar to glucose sensor systems. Further investigation is needed to extend biomarker analysis beyond metabolic detection, enabling comprehensive physiological monitoring and clinical decision-making.

Molecular sensor systems are used to detect and identify biological molecules due to their specificity and complexity (Figure 1b) [1,6,7,8]. However, advancements in synthetic recognition elements, viz., enzymes and polymers, have allowed for a level of selectivity comparable to natural ones. Conventional molecular sensor systems encompass all sensor systems designed to detect biologically significant molecules, emphasizing the importance of the analyte being detected rather than the specific recognition component utilized [1,9].

Molecular sensor systems generally consist of four primary components [10,11]: the analyte, representing the biomolecule targeted for detection; the recognition element, which engages with the analyte and imparts selectivity; transducer, responsible for converting the interaction between analyte and recognition element into a discernible signal; the analysis system, which interprets the acquired signals. Alongside these physical components, many essential terms are crucial for comprehending and contrasting sensing schemes. The limit of detection (LoD) is the minimum analyte concentration that a sensor system can reliably identify; sensitivity pertains to the sensor system’s capacity to distinguish between closely related input values. Selectivity refers to a sensor system’s capacity to specifically target particular analytes, whereas specificity, being the highest form of selectivity, denotes the sensor system’s ability to target a single analyte [12,13]. The linear range delineates the concentration spectrum of the analyte wherein a sensor system generates a proportionate response, whereas the physiological range refers to the concentration spectrum of the analyte that is physiologically relevant, preferably situated within the sensor system’s linear range [12,13].

Biological sensor systems use biological components, e.g., DNA, antibodies, and enzymes, to specifically recognize and bind to the target biomolecule, transforming this interaction into a measurable signal. Conversely, chemical sensor systems utilize inorganic or synthetic materials and rely on chemical reactions or the biomolecule’s properties to generate a signal. Based on these characteristics, we utilize the term multidomain molecular sensor systems to include both biological and chemical sensor systems. Previous reviews in this field summarizing the existing literature have also been restricted to biological-based sensor devices, often including surveys that investigate bodily fluid-integrated sensor systems.

This review examines multiple innovative approaches anticipated to advance the creation of multidomain molecular sensor devices. The review article explores current developments in multidomain molecular sensor systems, emphasizing the substrate materials, bodily fluids, power, and decision-making units to formulate a framework for the design and implementation of molecular sensor systems. A synopsis of the extensive molecular data acquired from multidomain molecular sensor systems in both normal and abnormal situations, utilizing recent computational algorithms for biomarker detection, is also presented.

## 2. Multidomain Molecular Sensor Devices

This section examines the advancement of multidomain molecular sensor devices, concentrating on two primary challenges: the shift from laboratory assays to in vivo systems for implantables or wearables, and the expansion beyond glucose detection to include a broader spectrum of physiological indicators.

### 2.1. Biological-Based Sensor Devices

#### 2.1.1. Signal Amplification Methods

**Figure 2:**
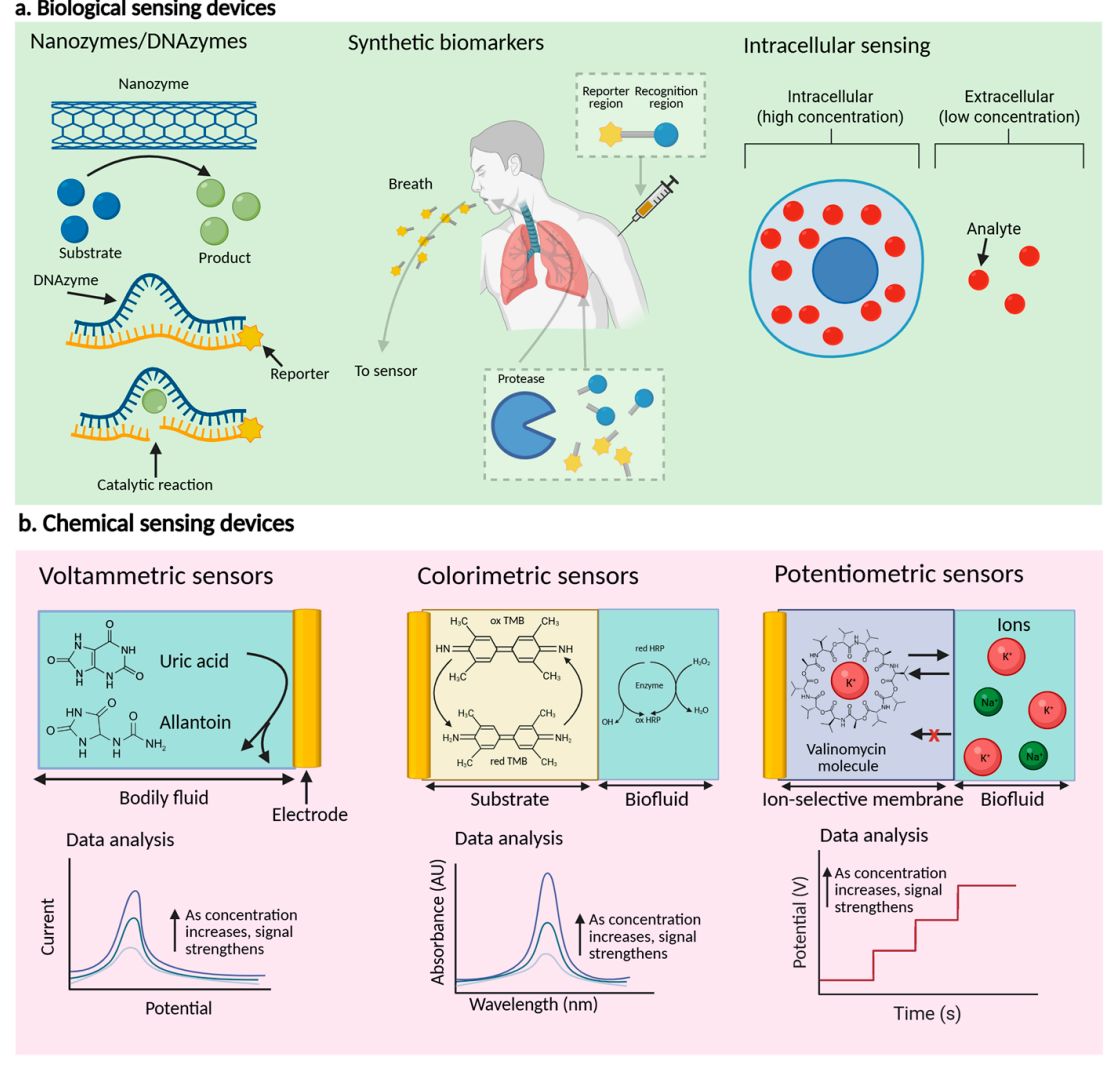
The main methodologies for signal transduction and recognition in different molecular sensor devices. (**a**) Methods for enhancing biological-based sensor devices. DNAzymes and nanozymes offer alternate approaches for monitoring analytes through catalytic processes. Synthetic biomarkers can be utilized to improve molecular activities and aid in their identification within the body. Intracellular sensing facilitates the identification of novel, high-concentration biomarkers. (**b**) Signal transduction and recognition for chemical-based sensor devices. Voltammetric sensor devices detect reduction or oxidation in an electroactive molecule, e.g., uric acid, by allowing electrons to be received on or released from the electrode’s surface, with the observed peak current magnitude directly proportional to the target analyte’s concentration. Colorimetric sensor devices utilize a substrate to immobilize a color indicator molecule, causing a color change when the analyte comes into contact. Horseradish peroxidase (HRP) detects hydrogen by altering the redox state of chromophore molecule 3,3’,5,5’-teraenthykbenzidine (TMB) during H_2_O_2_ enzymatic breakdown. The intensity of color change or light absorption corresponds to the substance being analyzed. Potentiometric ion-selective sensor devices utilize a working electrode with an ion-selective membrane, containing ionosphere molecules, e.g., valinomycin, which interacts with specific ions of specific size and charge, resulting in a potential that varies the target ion concentration. Figure created with BioRender (BioRender.com).

Biomarker analysis using conventional biological sensor devices encounters challenges in adapting to novel analytes, as well as issues with specificity and sensitivity. Various amplification techniques have been developed to achieve lower limits of detection and higher sensitivity, enabling practical physiological monitoring at any sensor development stage (Figure 2a) [13,24].

The limit of detection and sensitivity of traditional biological sensor devices rely on the amount of analytes interacting with the sensor’s recognition element [25,26]. Consequently, to ensure sensitive and fast detection, maximizing the analyte-recognition element interactions is necessary. Some strategies to tackle this limitation include synthetic nanochannels to maximize molecular localization, and molecular micromotors to accelerate mass transport and mix solutions [25,26]. Probe crowding is also an issue, resulting in the limitation of mass transport in conventional biological sensor devices; to resolve this, surface probe distribution can be improved.

The Debye length is the effective detection range and is often an obstacle for electronic sensor devices. To overcome this and amplify biological interactions, strategies, e.g., creating near-electrode conformational changes to signal distant interactions to accelerate electronic transfer and using membrane-encapsulated ion-free water layers to extend the Debye length, have been suggested [27,28]. The Debye length also serves as a threshold; surpassing it results in a “blind spot” where a sensor device is incapable of detecting the presence of an analyte due to the thermodynamic characteristics of charge distribution. To address this issue, researchers have also devised multiple strategies, including surface engineering and the modulation of the electric double layer.

Enzymes, viz., nanoenzymes and DNAzymes, are promising recognition elements because of their high selectivity and catalytic activity. However, traditional types of enzymes are limited to which analytes they can and cannot target.

Nanoenzymes are composed of nanomaterials and have a catalytic activity that replaces traditional enzymes in molecular sensor devices. These materials include nanoparticles and metal–organic frameworks (MOFs), which conduct biochemical catalytic reactions leading to amplified responses. For example, single-atom nanoenzymes maximize their catalytic activity by their atomically dispersed metal active sites, which could be selectivity-tuned, similar to natural enzymatic reactions [29,30].

DNAzymes catalyze reactions, e.g., RNA cleavage, RNA ligation, and alkyne-azide cycloaddition [31,32,33]. These reactions, combined with a catalytic event and release of a reporter molecule, can be utilized to detect metal ions and other small molecules. For instance, extending the sequence of DNAzyme to include an aptamer sequence to bind the target analyte has improved selectivity.

Synthetic biomarkers are artificially created molecules intended to identify and enhance disease-related signals, especially in the early stages of disease, by interacting with certain biological processes in the body. These biomarkers are artificially synthesized in a laboratory and engineered to improve the identification of diseases such as cancer, often by magnifying subtle molecular changes that would otherwise be difficult to detect. The synthetic biomarkers exhibit many designs, although all have two fundamental components: a recognition region to identify the biochemical activity and a reporter region to indicate their existence. For instance, peptides coupled to volatile reporter molecules, when injected into mice, are cleaved by neutrophil elastase, signifying its heightened activity, which correlates with various diseases.

Intracellular sensor devices offer specific information at the cellular level, surpassing membrane-bound and secreted biomarkers [34,35]. They have been utilized to identify overexpression of cytosolic cathepsin B in adenocarcinoma cells and to measure mRNA expression in breast cancer cells. These devices increase sensitivity due to the frequent occurrence of higher concentrations and the cell’s diminutive dimensions. They allow access to a wide range of biomarkers in high concentrations and facilitate cell identification by examining cytoplasmic regions [34,36,37,38]. Intracellular analysis is typically conducted through two methodologies: introducing a genetic sensor framework into the cell to activate reporter genes or inserting nanostructures into the cytosol to investigate its contents [34,35]. These approaches significantly enhance biological phenomena within cells, e.g., protein overexpression in the cytoplasm, for diagnosis purposes.

Similarly, multiplexing, i.e., using multiple markers, allows for the amplification of physiological signals by increasing the number of biological targets [29,34,35]. Multiplex controls, e.g., additional sensor devices with nonspecific recognition elements, allow for better sensor stability because they provide separate channels to monitor sensor degradation.

Another method to amplify biological interactions is to increase the amount of signal generated by an analyte [39,40]. The typical signal-to-interaction ratio of 1:1 can be increased by incorporating multiple reporter molecules in the recognition element. Additionally, a secondary reporter could be used to generate amplified responses; this reporter complexes with the oligonucleotide systems or the reporter-labeled nanomaterials. However, multimerization amplification is designed for traditional immunoassays and is less utilized in body-based biological sensor devices.

#### 2.1.2. Improvement Methodologies for Selectivity

Biological recognition elements can be incorporated into molecular sensor devices to improve selective detection of biologically relevant molecules [40,41]. Most biological recognition elements and targets do not have specific enzymatic catalysts, so high-affinity recognition elements are necessary for conventional body-based biological sensor devices. These recognition elements induce highly specific interactions with their target molecules [6,41]. They must withstand interferants, be stable during daily wear and tear, and exhibit the dynamic capability to measure relevant changes in biomarker levels. Traditional types of recognition elements have faced difficulties with nonspecific binding and biofouling, e.g., interferant accumulation. This section will discuss the solutions to these challenges and describe how these elements can be used for in vivo real-time monitoring.

Functional nucleic acids, viz., ssDNA/RNA, nucleic acid enzymes, aptamers, and SOMAmers, exhibit great potential as affinity recognition elements [42,43]. Aptamers are single-stranded nucleic acids created in vitro from systematic evolution of ligands by exponential enrichment (SELEX), in which sequences that bind to specific targets are selected [42,44]. These targets are on a broad range, e.g., toxic, non-immunogenic, and small-molecule biomarkers, which are difficult for antibodies to target. Aptamers have promising implementation value in body-based biological sensor devices because of their inexpensive and facile mass production, high thermal stability, and site-specific chemical modification ability. However, conventional types of aptamers have a negative charge and can be prone to nonspecific binding. They are also susceptible to nucleases in biological fluids and are sensitive to changes in environmental conditions, i.e., the pH. SOMAmers, which are chemically modified aptamers with non-natural nucleic acids, can be utilized [41,44]. These substitutes have low nonspecific binding, improved stability, and higher binding affinity than traditional variants of natural aptamers.

Antibodies are highly utilized natural affinity receptors in biological sensor devices [6,45]. Their immunostimulation is specific due to their selectivity when binding targets; they have high binding affinities, selectivity, and sensitivity to biomarkers. However, conventional types of natural antibodies are fragile and expensive to generate. Therefore, artificial antibody mimetics are designed to address the limitations of conventional natural antibodies. Antibody mimetics can be generated to have a high ligand-binding affinity; they tend to be smaller, have higher thermal stability, and display increased avidity compared to their natural counterparts [41,45]. Prototypical varieties of these engineered antibodies, although a great alternative, still have their constraints as well. They cannot fully replicate natural antibodies and fail to outcompete them in assay performance, likely due to their inability to mimic glycosylation patterns.

One challenge for selectivity improvement is nonspecific binding, which occurs as biofouling on the sensor surface and as cross-reactivity on the recognition element [41,45,46,47]. Biofouling is the accumulation of nonspecific binding with other cells, molecules, or species on the sensor surface; this accumulation hinders the diffusion of traditional analytes and leads to signal loss [46,47]. Cross-reactivity occurs when traditional recognition elements interact with molecules other than the target analyte to generate false-positive results [10,41]. To combat biofouling and cross-reactivity, techniques and strategies, e.g., utilizing protective coatings and exploiting kinetic differences between analytes and their interferents, should be developed.

To exploit the differences between the analyte and the interference, three strategies can be observed [42,48]. First, weak-bound interferants can be extracted with an external force, e.g., the alternating electric field. Second, a reference or control sensor can be included with a nonspecific receptor. Third, a stochastic sensory approach that involves computational distinction between the target analyte and the interferant. Most of these strategies help improve resistance to nonspecific behavior and render sensor devices less prone to nonspecific interactions.

Anti-fouling is a strategy that aims to fill gaps in the sensor surface using a blocking agent or coat the entire sensor surface with an anti-fouling layer [44,49,50]. Examples of possible blocking agents include bovine serum albumin (BSA) and casein. Some anti-fouling layers incorporate poly(ethylene glycol) and zwitterionic polymers.

#### 2.1.3. Regentless, Real-Time Continuous Monitoring

To develop a promising candidate for body-based sensor devices, biological sensor devices should be able to detect analytes using reversible interactions and provide continuous data [51,52]. The receptors of these sensor devices should have fast binding kinetics that allow rapid equilibration with the surrounding fluid for continuous monitoring. However, faster binding kinetics come at the expense of the traditional sensor device’s limit of detection. To combat this issue, biological sensor devices should utilize susceptible transduction mechanisms, e.g., DNA-based methodologies, to compensate for sensitivity loss. Continuous monitoring can be achieved when recognition elements are regenerated after each measurement [13,52].

Continuous monitoring requires complementary sensor techniques [1,53]. Conventional sensor methodologies utilize secondary reporters, indirect reporters, or chromatographic systems; however, they are incompatible with body-based biological sensor devices because they require external manipulation. To achieve excellent body-based sensor devices, external modification should not be required, and detection methods should have versatile target analysis and reagentless mechanisms [1,53]. There are numerous promising reagentless and real-time continuous body-based biological sensor devices, including electrochemical aptamer-based sensor devices, electrochemical DNA sensor devices, DNA scaffold-based sensor devices, and protein scaffold-based sensor devices [54,55,56].

Protein scaffold-based sensor devices operate through reagentless detection of the specificity of amino acid scaffolds [1,57]. These protein scaffolds comprise an electroactive reporter that confers electrochemical activity, in which the protein scaffold itself functions as the analyte receptor. Due to this, a separate recognition element is not required. However, dependence on protein-based receptors greatly limits the range of analytes traditional protein-based sensor devices are able to detect.

Electrochemical DNA (E-DNA) sensor devices operate through target-induced proximity changes between the terminal redox reporter and the electrode [58,59]. They depend on DNA hairpins that hybridize to nucleic acid analytes. They also employ strand displacement techniques. These devices can be favorably utilized in in vivo systems for detection in whole blood [1,57]. However, traditional E-DNA sensor devices generate false-positive results because of ultrasmall concentrations of circulating DNA in physiological matrices, which complicates the commercial adaptation of these devices.

DNA scaffold-based sensor devices operate through reagentless detection of proteins, nucleic acids, and small molecules [59,60]. These devices utilize an array of small recognition elements that are anchored to electrodes by DNA sequences. They compare bound versus unbound probes to differentiate the faradaic current. However, conventional DNA scaffold-based sensor devices are incompatible with larger recognition elements, viz., antibodies; they exhibit slow diffusion and low baseline currents.

Electrochemical aptamer (E-AB) sensor devices detect small molecules and proteins through reagentless and label-free detection. These devices utilize electrode-bound aptamers to recognize and bind analytes. Analyte detection is achieved through target-induced proximity changes between the redox reporter and the electrode surface. E-AB sensor devices are optimized to have a detailed theoretical framework, demonstrate an excellent temporal resolution in in vivo measurements, are implantable, and monitor non-invasively and continuously [54,55,56]. The conventional E-AB sensor devices’ main limitations are that they are restricted by the availability of aptamer sequences that bind relevant targets and have conformational changes between their unbound and bound states.

### 2.2. Chemical-Based Sensor Devices

#### 2.2.1. The Principal Recognition and Signal Transduction Methodologies

Chemical-based sensor devices are one of the promising candidates used for body-based sensor devices, as they perform in a reagentless and continuous manner (Figure 2b) [61,62]. The major types of these devices are voltammetric sensor devices, colorimetric sensor devices, potentiometric sensor devices, amperometric sensor devices, and fluorescence sensor devices, based on signal transduction mechanisms [63,64]. The principles, advantages, applications, and limitations of these devices are examined to comprehensively understand their functionalities and potential in various applications.

Voltammetric sensor devices involve varying the applied potential with controlled steps and speeds [65,66,67]. Various techniques, e.g., cyclic voltammetry, differential pulse voltammetry, and square wave voltammetry, are utilized. Differential pulse voltammetry and square wave voltammetry minimize background charging currents, resulting in highly sensitive measurements. Stripping voltammetry is particularly effective for detecting ultralow levels of heavy metals in bodily fluids.

Colorimetric sensor devices rely on chromophore molecules that utilize color indicators [68,69]. External stimuli alter the chromophore, affecting light absorption. These devices can be coupled with enzymatic reactions to target specific recognition events. The measured color intensity correlates with the target analyte’s concentration. Qualitative analysis can be conducted visually or with a camera.

Potentiometric sensor devices utilize the potential difference between working and reference electrodes [70,71]. Solid-state ion-selective electrodes are used in molecular sensor devices to assess electrolytes or ions in biofluids. The working electrode is enhanced with an ion-selective recognition membrane, allowing interaction only with certain ions. This selective recognition process generates an electrochemical phase boundary potential, which is converted into a voltage signal by a transducer. The potential signal between the reference electrode and the ion-selective electrode exhibits a log–linear correlation with the concentration of the target ion, as described by the Nernst equation.

Amperometric sensor devices operate by applying a potential that facilitates the redox reaction of an electroactive molecule [72,73]. The current generated by the electron transfer between the molecule and the electrode serves as the signal. The incorporation of enzymes enhances selectivity, leading to the development of amperometric enzymatic detection. In these devices, the sensor electrode is modified by an enzyme, allowing continuous measurements with fast response times. This technology is widely utilized in commercial continuous glucose monitoring.

Fluorescence sensor devices utilize fluorophores for signal transduction, which absorb light and emit it at longer wavelengths [68,74]. Ionochromism alters the fluorescent light signal’s intensity. These devices require a light source and optical accessories to obtain accurate quantitative luminescent outputs and are highly sensitive.

#### 2.2.2. Methods for Improved Biomarker Detection

Advancements in chemical sensor technologies have paved the way for innovative applications in medical diagnostics, environmental monitoring, and personalized healthcare [75,76]. To enhance the performance and functionality of chemical-based sensor devices, there are crucial material considerations to factor. These include skin conformability, electrode surface, surface coatings, and electrode materials [77,78].

Anti-biofouling protective hydrogels and polymeric coatings are essential for extending the stability of chemical-based sensor devices, particularly in continuous molecular profiling [49,79]. These coatings resist the adhesion of fouling or interfering molecules onto the electrode, maintaining the chemical sensor’s accuracy and longevity.

For chemical-based sensor devices, conformal contact with human tissues is crucial [76,80]. This requires materials that match the skin’s mechanical properties, e.g., soft and stretchable elastomers. However, there is a trade-off between higher conductivity and greater stretchability, necessitating an optimal ratio of both to ensure effective performance.

The transducers in chemical-based sensor devices predominantly utilize conducting carbon or gold materials due to their stability, conductivity, and ability to anchor recognition elements, e.g., self-assembled monolayers [75,76]. Among the most effective materials are nanomaterials, viz., carbon-based nanomaterials, which offer enhanced electron transfer and signal amplification.

## 3. Multidomain Molecular Sensor Systems

**Figure 3:**
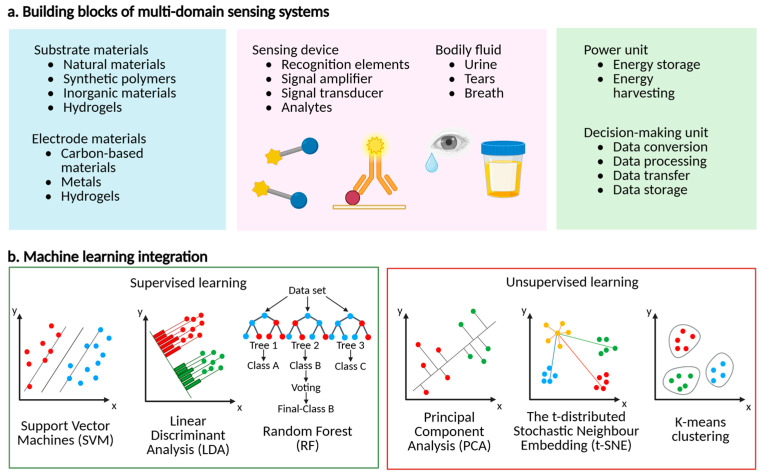
Summary of multidomain sensor system’s fundamental components and multidomain molecular sensors’ data-driven procedure and algorithm overview. (**a**) Body-based molecular sensor systems consist of substrate and electrode materials, sensing devices, bodily fluids, power and decision-making units, which are the fundamental components. (**b**) The principles of three exemplary supervised and unsupervised learning algorithms. Schematic illustrations of support vector machines, linear discriminant analysis, and random forest algorithms for addressing classification problems. Schematic representations of principal component analysis, t-SNE clustering techniques for the reduction in data dimensionality. Schematic diagram of k-means clustering for data cluster analysis. Figure created with BioRender (BioRender.com).

Multidomain molecular sensor systems utilize multiple components to provide optimal and effective biomarker detection (Figure 3a) [81]. This section will examine the key system components, viz., substrate materials, bodily fluids, power units, and decision-making units.

### 3.1. Substrate Materials

Substrate materials for multidomain molecular sensor systems can be broadly categorized into natural materials, synthetic polymers, inorganic materials, and hydrogels [82,83]. Hydrogels, which can be natural or synthetic, exhibit distinct properties that render them appropriate for molecular sensor systems. They are highly biocompatible, soft, deformable, transparent, and biologically friendly owing to their hydrophilic characteristics and porous networks that allow for substantial water retention. Hydrogels are utilized in molecular sensor systems for mechanical and chemical detection, although conventional hydrogels exhibit deficiencies in mechanical properties, e.g., flexibility, toughness, and continuous robustness. Additionally, conventional hydrogels are costlier than other substrate materials.

Natural materials, such as cotton, wool, linen, and silk, are derived from biological sources and are esteemed for their mechanical strength, flexibility, comfort, biocompatibility, and sustainability [84,85]. However, conventional natural materials often possess some deficiencies in desired physical properties, e.g., conductivity and optical attributes. To overcome these limitations, natural materials can be integrated with other substrate materials to generate mosaic materials, incorporating optical fibers with probing fabric materials or large-scale digital knitting textiles [86,87].

Synthetic polymers provide a diverse array of fabrication methodologies, including weaving, molding, lamination, photolithography, milling, and 3D printing [88,89]. These polymers are valued for their mechanical properties and are used to generate sensor system components, e.g., layer-by-layer assembled semi-flexible circuitries, stretchable substrates, and textiles. Synthetic polymers have been utilized to tune the mechanical and hydrophobic properties of fabrics, rendering them tough, flexible, waterproof, and breathable. The majority of molecular sensor systems consist of synthetic polymers, which operate as the primary flexible substrate that maximizes performance.

Inorganic materials, such as metals, semiconductors, and nanomaterials, are known for their high conductivity and excellent mechanical properties, viz., flexibility and elasticity [90,91]. Advanced fabrication techniques for inorganic materials include metal printing, nanomaterial ink printing in serpentine patterns, and weaving metal threads. The integration of inorganic materials into molecular sensor systems is achieved through layer-by-layer strategies, creating flexible, complex multilayer electronic applications, e.g., ultrasound transducers [92,93]. Graphene, a highly conductive material with good mechanical properties, is utilized for molecular strain sensor systems, printed circuit paths, capacitors, and transistors. However, the insufficient biocompatibility of traditional inorganic materials, viz., nanomaterials, presents biohazard risks.

### 3.2. The Bodily Fluids

Multidomain molecular sensor systems have progressed significantly due to advancements in non-invasive and bodily fluid-based technologies [94,95]. Various bodily fluids, e.g., urine, tears, breath, blood, saliva, sweat, interstitial fluid, and digestive fluids, are being utilized for at-home and continuous monitoring [96,97]. This section examines the potential and limitations of various bodily fluids for biomarker detection.

Urine serves as a reliable fluid for at-home molecular sensor systems owing to its non-invasive characteristics and simplicity of collection [98,99]. Molecular data from urine exhibit a strong correlation with blood concentrations, providing a less invasive methodology for monitoring. Furthermore, urine offers a long protein lifetime and stability, rendering it suitable for prospective molecular sensor systems [99,100]. However, the biomolecules’ concentration in conventional urine is generally lower than that in blood, and the prolonged filtration process during urine generation may hinder the timely identification of changes.

Tears provide a non-invasive monitoring technique, demonstrating a connection with blood glucose levels that entails a lag time of around 10 min [101,102]. However, the limited sample volume and rapid evaporation rates of conventional tear modalities hinder the efficacy of molecular sensor systems. Furthermore, secretion variations influence the consistency of data collected from traditional tear types.

Breath comprises volatile organic compounds, proteins, fatty acids, and other biomarkers essential for physiological and disease detection [103,104]. Molecular sensor systems, e.g., masks, analyze exhaled air to detect small molecules. However, the variety and abundance of biomarkers in conventional breath samples are generally lower than those observed in other bodily fluids, hence constraining their applicability.

Digestive fluids, analyzed using consumable electronics, e.g., diagnostic pills, provide a wealth of biochemical information related to health [105,106]. Prototypical digestive fluids are distinguished by strong acidity and a plethora of enzymes, necessitating robust molecular sensor systems. Digestive fluid analysis of the gastrointestinal (GI) tract is enhanced, for instance, through the detection of gastrointestinal bleeding using wireless optical-readout capsules.

Interstitial fluid (ISF) is a promising bodily fluid for biomarker detection due to its ability to collect non-invasively [107,108]. Alterations in analyte levels can be promptly identified in interstitial fluid, with a lag time of approximately 5–6 min for glucose. However, challenges such as analyte partitioning, physiological correlation, and lag times associated with traditional interstitial fluid need to be addressed for accurate detection.

Saliva is readily obtainable and easily stimulated, rendering it an advantageous bodily fluid for biomarker detection [109,110]. It possesses a diverse molecular composition and can be monitored continuously using body-based molecular sensor systems, e.g., mouthguards. Saliva-based diagnostics hold significant potential for infectious diseases. Nevertheless, prototypical saliva types necessitate independent validation and testing to ascertain the accuracy and reliability of the gathered data.

Blood is one of the most information-dense fluids for body-based molecular sensor systems [111,112]. It provides a comprehensive overview of the body’s biochemical condition. However, traditional methods of blood collection are invasive and present hazards of infection. Moreover, the high concentration of proteins leads to biofouling, decreasing the stability and reliability of molecular sensor systems over time.

Sweat is a non-invasive fluid that parallels blood in molecular concentrations, exhibiting a delay time of approximately ten minutes for glucose levels [113,114]. Traditional molecular sensor systems have been developed to detect small molecules in sweat, although the detection of larger molecules remains challenging. The effectiveness of conventional sweat-based sensor systems depends on sweat rate and analyte dilution, and there is a deficiency of biomarker-focused sweat proteome investigations.

Each bodily fluid presents unique advantages and challenges for molecular sensor systems. The fluid selection is dependent on the specific application, desired sensitivity, and invasiveness level [115,116]. With technology advancements, the integration of these fluids into molecular and at-home sensor systems offers potential for enhanced and non-invasive health monitoring.

### 3.3. The Power Units

The energy consumption of multidomain molecular sensor systems is dependent on the application and materials utilized in their construction. These systems typically require a power unit to generate electrical voltage and occasionally extract energy from the environment or the body. Ideal power units should be non-toxic, small, recyclable, and capable of harvesting energy or offering high energy density for an extended period [117,118]. Energy harvesting for these systems can be achieved through various methods, e.g., piezoelectricity, triboelectricity, thermoelectricity, optoelectronics, electromagnetic radiation, and catalytic reactions. These methodologies facilitate self-powered sensor systems powered by biomechanical, electromagnetic, biochemical, or a mix of mechanisms.

Photovoltaic materials are utilized for solar-powered molecular sensor systems [119,120]. Although traditionally rigid, flexible photovoltaic harvesters have been developed based on smart textiles composed of soft composite materials, allowing for photorechargeable power sources and washability.

Thermoelectric generators, which convert small amounts of heat into electricity, serve as an additional energy source for molecular sensor systems [117,118,121]. These generators utilize heat generated by the human body and, when combined with hybrid organic-inorganic materials, are well-suited for flexible sensor systems. However, prototypical thermoelectric generators exhibit lower power densities and energy conversion rates compared to piezoelectric and triboelectric generators.

Piezoelectric and triboelectric phenomena transform uneven mechanical energy into electricity [122,123]. Generators utilizing these effects, with stretchable electrodes and adjustable dimensions, can be affixed to the skin and provide high output voltage and stability over many operating cycles [121,123]. However, conventional triboelectric generators encounter challenges regarding longevity. The design, miniaturization, and manufacturing of piezoelectric and triboelectric harvesters are crucial for maintaining stability in molecular sensor systems.

Another promising energy source is the biofuel cell, which utilizes enzymes as catalysts to convert chemical energy into electricity [124,125,126]. For example, lactate drives self-powered molecular sensor systems through oxidation by oxidase enzymes. Challenges with biofuel cells include increasing energy density, stability, longevity, and catalyst efficiency, as well as limited fuel availability and miniaturization. Nanoenzymes and nanotubes are promising alternatives for catalyzing biofuel cells and enhancing efficiency, with significant potential for on-skin biofuel cells in molecular sensor systems [127,128].

### 3.4. Decision-Making [122,123] Units

The decision-making unit in multidomain molecular sensor systems plays a crucial role in converting raw physiological data into a human-readable format [11,124]. This unit accesses physiological information through distributed sensor arrays, creating a comprehensive database that spans from individual users to larger populations. The primary function of the decision-making unit is to extract patterns and relationships from the data while optimizing the potential of data fusion [124,125].

The decision-making unit’s operation begins with distributed sensor systems translating physiological data into digital signals, which initiate the data pipeline [129,130]. Raw data are sent to a data conversion component, where digital signals are transformed into secondary data using corresponding algorithms [129,130,131]. This transformation is expressed through a conversion function that correlates the digital signal with the biomarker, determined by regression analysis. This process may necessitate additional assumptions that are not immediately apparent to the downstream application, together with data filtering, smoothing, denoising, or downsampling.

After the secondary data are prepared for the downstream model, various procedures are conducted, e.g., outlier and anomaly detection, data clustering, noise reduction, handling of missing features, data normalization, dimensionality reduction, and baseline correction [130,131].

Feature engineering is then utilized to maximize the relevant information density from the high-dimensional data for the given task, discarding less useful components to avoid encumbering the learning process [130,131]. This involves combining secondary data features into new variables, reducing dimensionality while conserving data variance, and transforming coordinates. The interpretation of these engineered data is also connected with other information types within the data pipeline.

An automated model designed to find patterns and achieve specific goals is fed with high-dimensional data, enhanced with engineered features [124,125]. This model is typically utilized for tasks, e.g., classification, regression, clustering, or reducing data dimensions, depending on whether the data are labeled. The labels, which provide important information about the body’s hidden physiological state, are either categories or numbers assigned by humans.

In supervised learning, the model is trained using the labelled examples to predict the body’s state [125,132]. However, the model’s accuracy depends on the label quality and any biases from the human-supervised process. It is important that training examples represent the entire population of interest, that there are sufficient examples to reduce noise, and that training strategies consider imbalances, e.g., disease prevalence. Additionally, the confidence in the labels’ accuracy, i.e., “ground truth”, should be boosted through multiple expert opinions [125,132].

Data-driven learning can be challenging due to the existence of multiple models with differing complexities that address the same issue. To minimize errors, the model’s complexity has to correspond with the type and amount of data. An effective approach is ensemble learning, which integrates different models employing various learning methodologies to facilitate decision-making [133,134].

In healthcare, model complexity is especially important because it pertains to detecting individual health patterns. For instance, detecting epileptic seizures from brain signals is difficult due to insufficient data, rendering simpler models, e.g., support-vector machines or random forests, more effective than deep neural networks [133,135]. In small datasets, conventional deep neural networks memorize patterns instead of learning from them, leading to inaccurate predictions with new data. However, for more predictable conditions, viz., arrhythmia, larger datasets can be utilized to train more complex models.

The model’s core algorithm should consider the specific nature of the problem [10,133]. For example, the COVID-19 model was developed to incorporate multiple physiological markers, e.g., muscle deterioration, respiratory changes, and vocal cord differences, to enhance sensitivity in disease detection.

The model’s output can be generated in a discrete manner or continually, contingent upon its intended function [131,132]. An essential aspect of the design process is determining data management strategies. Model outputs can be transferred between molecular sensor systems, their software, and external technologies, e.g., smartwatches or smartphones [131,136]. This allows for real-time recording and wireless transfer of important health data. The data transmission methods are dependent on the anticipated energy consumption and the application type, i.e., whether it involves real-time or one-time analysis. In the future, wireless methodologies, viz., Bluetooth and LoRa, could allow fast, short, and long-distance transmission, while optimizing energy usage. Currently, radio transmission utilizes more energy than local data processing. To conserve energy, future systems should prioritize data processing and decision-making at the sensor device level [136,137].

For molecular sensor systems, data storage is important. There are two primary categories: volatile memory, which impacts system performance and energy consumption, and non-volatile memory, which consumes less energy but has slower data transfer rates [10,138]. Optimal performance can be attained by utilizing both memory types, alongside meticulous design of data collection frequency, sensor integration, and energy management.

## 4. Algorithms for Multidomain Molecular Sensor-Facilitated Data-Driven Biomarker Detection

The progress of artificial intelligence and bioinformatics has resulted in increased interest in machine-learning-based big data disease diagnosis, owing to its cost efficiency and high precision [139,140]. Machine-learning algorithms are distinctive in addressing issues, e.g., regression and classification, via autonomous learning from datasets. In disease diagnostics, machine learning facilitates the extraction of logical principles from intricate disease data, creating training models capable of identifying, classifying, or predicting unknown samples. Comprehending the concepts of these algorithms improves interpretability and provides a theoretical basis for the selection, analysis, and interpretation of illness diagnostic models. In this section, machine-learning algorithms will be classified into supervised and unsupervised learning based on the labelling of the training data.

### 4.1. Supervised Learning Algorithms

Supervised learning is a technique that uses existing datasets as a training set to develop a mathematical model by discerning the link between labels and features. This facilitates precise predictions while handling unlabeled sample data. Supervised learning is classified into classification and regression tasks according to the discrete or continuous character of the output data. Regression problems focus on estimating true values, while classification problems include forecasting sample classes. The distinction between classification and regression is ambiguous, since continuous data in regression may be converted into classification tasks. Diverse supervised learning methods, e.g., support vector machines (SVM), linear discriminant analysis (LDA), random forest (RF), and neural networks (NNs), are extensively used for disease diagnosis [141,142].

Support vector machines (SVMs) are a binary classification approach that improves the generalization capability of mathematical models by maximizing the margin between two classes [143,144]. When faced with linearly divisible problems, researchers seek to ascertain if a straight line may efficiently categorize data into discrete classes. It is extensively used in disease diagnostics owing to its capacity to formulate decision functions from minimal sample data. For example, researchers have created ratiometric 3D DNA probes for the ultrasensitive fluorescence detection of numerous biomarkers in urinary extracellular vesicles (uEVs) with k-nearest neighbors and support vector machine methods. The optimum biomarker combination attained diagnostic accuracy values of 95% and 100% when assessed using k-nearest neighbors and support vector machines, respectively.

Linear discriminant analysis is a traditional machine-learning technique that maps data from a specified training dataset onto a linear subspace, with the objective of enhancing the closeness of the data points within the same category while maximizing the distinction between projected points of different categories [145]. It demonstrates efficient dimensionality reduction and classification abilities, along with resilience to noise interference. For instance, researchers have generated a nanoenzyme sensor array for ultra-sensitive ratiometric fluorescence detection of exosomal proteins, with a diagnostic accuracy of approximately 100% [146].

Random forest is an ensemble of several decision trees, with each tree operating as an independent classifier. It exhibits insensitivity to default parameters and demonstrates reduced susceptibility to overfitting [147]. For example, researchers have created urinary multi-molecule electrochemical sensor systems and evaluated the multi-molecule signals using random forest and neural network algorithms. The researchers observed that random forest algorithms surpassed neural network algorithms due to their ability to provide interpretable values and emphasize the significance of specific biomarkers, a crucial factor in developing a biomarker panel with few biomarkers. With appropriate biomarker combinations, random forest achieved 100% accuracy in detecting prostate cancer patients within a group of approximately 20 people [141].

### 4.2. Unsupervised Learning Algorithms

Unsupervised learning algorithms are used to derive representative features from unlabeled datasets and to graphically depict them. They are primarily utilized in two areas: mitigating data sparsity and complexity via dimensionality reduction techniques, e.g., principal component analysis (PCA) and t-distributed stochastic neighbor embedding (t-SNE), and categorizing data points based on pairwise similarity metrics through clustering methods, viz., k-means clustering and hierarchical clustering [148,149].

Principal component analysis significantly reduces dataset dimensionality by retaining low-order principal components and disregarding higher-order ones, hence preserving characteristics with the greatest variance [150]. For example, researchers have used principal component analysis to examine surface-enhanced Raman scattering characteristics of blood samples from renal cell cancer patients and controls. Their results indicated a propensity for unsupervised clustering between the two groups, establishing a basis for supervised learning algorithms to differentiate renal cell cancer patients from controls [151].

The t-distributed stochastic neighbor embedding is a nonlinear dimensionality reduction approach that converts the similarity of data points into conditional probabilities, facilitating the mapping of high-dimensional data to low-dimensional space while maintaining local features for exploration and visualization [152]. For instance, researchers have used t-SNE analysis on mass spectrometry images of several tumor subpopulations to elucidate statistical correlations between tumor subpopulations and patient survival in gastric cancer, as well as metastatic status in primary breast cancer tumors [153].

The k-means clustering is an algorithm that groups very similar data by measuring the distance between them [154]. It provides benefits, e.g., rapid computing efficiency, ease of understanding, and simplicity. For instance, a DNA photonic wire was created for the concurrent detection of two miRNAs markedly elevated in individuals with acute myocardial infarction (AMI). The outcomes were then grouped by k-means clustering, yielding a 100% correct diagnosis of early acute myocardial infarction in both the training and validation cohorts of clinical blood samples [155].

### 4.3. Machine Learning for Disease Diagnosis Applications

Machine learning for disease diagnosis applications has garnered interest in several disciplines, e.g., chemistry, biology, and medicine. As domains and application requirements converge, these applications have developed into two modular workflows: (1) direct diagnosis via machine learning and (2) diagnosis using machine learning and validation. The distinction between these applications arises in the diagnostic and data collection modules.

The machine-learning procedure for direct diagnosis entails screening several biomarkers for a particular disease, gathering data, training using appropriate algorithms, and assessing the outcomes using established medical assessment criteria. This modular technique facilitates rapid screening, categorization, or diagnosis of the disease [156,157].

Machine-learning-based direct diagnosis utilizes biosensing assays for data collection. For example, researchers established a quantitative analytical platform using the DNA-Paint technique to assess various exosome surface biomarkers at the individual exosome level. The researchers used linear discriminant analysis and cross-validation to develop biomarker profiles for quick disease diagnosis. The researchers effectively used this technology to identify exosomes in blood samples from cancer patients, attaining a 100% accuracy rate for pancreatic and breast cancers in unidentified samples. Researchers also used microfluidics to conjugate DNA aptamers, e.g., EpCAM and HER2, to the surface of extracellular vesicles [158]. The researchers then separated specific subpopulations of vesicles using λ-DNA-mediated viscoelastic microfluidics. Two-dimensional analysis of individual vesicles was conducted based on size and label expression. Employing linear discriminant analysis and t-distributed stochastic neighbor embedding, distinctive signals were detected inside vesicles, successfully differentiating several breast cancer cell lines and patients with differing HER2 expression levels [159].

The diagnosis using machine learning and validation entails the selection of many biomarkers for a particular disease and the acquisition of biomarker data from both training and validation cohorts [160,161]. The training cohort data are used to train the machine-learning models, which are then verified using independent validation cohort data. The outcomes are assessed via normal medical assessment criteria.

Multiple omics testing methodologies have been used to perform diagnosis using machine learning and validation [162]. For instance, researchers used an electrochemiluminescence immunoassay, droplet digital polymerase chain reaction (PCR), next-generation sequencing, quantitative polymerase chain reaction, and a track-etched magnetic nanopore device to enhance the detection and staging of pancreatic ductal adenocarcinoma (PDAC). The training cohort’s multiomics data were trained with classifiers, e.g., I Bayes, logistic regression, linear discriminant analysis, support vector machines, and k-nearest neighbors. The findings were averaged to develop a diagnostic or staging model for PDAC, which was then validated using an independent validation cohort. The diagnostic model attained an area under the curve (AUC) of 0.95 and an accuracy of around 90%, whilst the staging model exhibited an accuracy of approximately 85% [163].

The diagnosis using machine learning and validation has also been created using biosensing assays [164]. For example, researchers used fluorescent aptamers to tag proteins on extracellular vesicles and utilized a thermophoretic aptasensor (TAS) for enrichment purposes [165]. A cancer detection and classification model was developed with a two-step linear discriminant analysis on a training sample of around 70 individuals. The first linear discriminant analysis distinguished between cancer patients and healthy controls, but the subsequent linear discriminant analysis used gender and seven indicators to categorize various cancer types. Performance assessments were examined with t-distributed stochastic neighbor embedding. The trained model was then used on a validation cohort of approximately 100 individuals across six cancer types at stages I-IV, attaining a specificity of 100% and a sensitivity of 95% for identifying stage I cancers, with an overall accuracy of approximately 70% in categorizing diverse cancer types. This test illustrates the promise for rapid and economical cancer screening, classification, and monitoring methods.

In the case of limitations, traditional direct diagnosis by machine learning is applicable to the majority of molecular sensor systems and has considerable adaptability [166]. However, its diagnostic accuracy is intricately linked to sample size and needs improvement [167]. A solitary sample diagnosis is challenging. The conventional diagnosis using machine learning and validation may enhance diagnosis accuracy via cross-validation of training and validation cohorts; nevertheless, it requires a substantial volume of samples and data, and the costly and intricate processes of sample collection and testing restrict its broader use.

## 5. Conclusions

The SARS-CoV-2 pandemic has generated heightened interest in molecular sensor systems, with the utilization and rapid advancements of viral antigen tests demonstrating the sensor systems’ effectiveness in providing health-related data. Previously, glucose monitoring was the primary focus, but now there’s a growing interest in developing multidomain molecular sensor systems for various analytes in different bodily fluids.

Body-based molecular sensor systems require biocompatible and flexible electronic materials that can be easily connected with soft biological tissues. These materials can be created using scalable, simple, and inexpensive technologies. Organic materials and polymers offer biodegradability, flexibility, and cost-effectiveness, while conductive hydrogels with laser-engraved graphene function as the sensor platforms. Fluid metals, viz., gallium, exhibit significant potential for soft electronics advancement. Material considerations depend on the application, e.g., consumable sensor systems made from biosafe, edible materials, implantable sensor systems fabricated from biofilm-resistant materials, and molecular sensor systems in smart textiles. Materials capable of facilitating multi-sensor communication, storing energy, and extracting biomechanical or biochemical energy are also crucial for the advancement of body-based molecular sensor systems.

Molecular sensor systems in clinical settings require safety demonstrations. They have to be comfortable to wear, generate minimal irritation, exhibit low immunogenicity and toxicity, and be sanitized. Systems should be resilient against intrusive incidents and cyberattacks while ensuring user data protection. These systems should also be resistant to light and noise, and generate minimal stimuli.

Body-based molecular sensor systems are revolutionizing personalized healthcare by providing real-time management plans, interventions, and diagnoses based on patient-specific biomarker data. These systems detect early illness indications, supervise drug dosages, and determine effective medications. They also offer proactive insights, e.g., nutritional and metabolic profiles, to prevent diseases and improve patient well-being. Body-based molecular sensor systems have the potential to develop sophisticated closed-loop systems, viz., artificial pancreas systems, for diabetes management and treating health conditions associated with biomarker deficiencies, i.e., iron-deficiency anemia and neural chemical imbalances.

Advanced molecular sensor applications aim to improve biomarker analysis by using sophisticated body-based molecular sensor systems for detecting various analytes in biological environments. However, significant obstacles have to be addressed, e.g., ensuring practical and reliable system integration, mitigating interferences in complex fluid compositions, developing regenerable sensor approaches, and improving signal amplification. These obstacles need to be alleviated for widespread adoption.

Molecular sensor-based biomarker detection involves collaboration between medics, engineers, biologists, and chemists to create integrated, advanced molecular sensor systems capable of accurately detecting and forecasting impending events. This technology will enable instantaneous analysis of genomics, proteomics, metabolomics, and other omics, allowing for prompt therapies, and early disease diagnosis and prediction [168,169,170,171]. The vast amount of data gathered from extensive studies are crucial for this research area.

## Data Availability

No primary research results, software or code have been included and no new data were generated or analyzed as part of this review.
